# Notes and News

**Published:** 1905-08-05

**Authors:** 


### NOTES AND NEWS.

It is proposed to establish a convalescent home in connection with the Great Northern Central Hospital. This will enable the hospital to receive a considerably larger number of patients.

The medical superintendent of the Montrose Asylum mentions motoring as the latest addition to the list of predisposing causes tending towards insanity. He reports two victims of the new "motor mania" at his institution. He gives no details to enable us to determine what form the craze assumed, or whether the tendency was homicidal, suicidal, or of a less personal character. But in any case the new menace to mental stability is disquieting.

At the opening meeting of the Council of the Liverpool Institute of Tropical Research, which was held on Monday, the chairman, Sir Alfred Jones, emphasised the necessity for applying scientific methods to commercial enterprises in the tropics. In some respects, he thought, this necessity is more widely recognised in Germany, France, and Belgium than in Great Britain. He guaranteed the Institute £1,000 a year for four years. The following additional guarantees were announced:—Mr. W. H. Lever, £1,000 a year for four years; Mr. T. Sutton Timmis, £250 a year; Mr. H. Sutton Timmis, £50 a year.

The first Congress of the International Society of Surgery will be held at Brussels from Monday, September 18th, until the following Saturday. Dr. Theodor Kocher, professor of surgery in Berne, will act as president of the Congress, which is under the patronage of the King of the Belgians. The following subjects will be discussed: The Value of the Examination of the Blood in Surgery; the Treatment of Prostatic Hypertrophy; Surgical Intervention in Non-Cancerous Diseases of the Stomach; Treatment of Articular Tuberculosis; and the Diagnosis of Surgical Diseases of the Kidney. Information can be obtained from the English delegate, Mr. Reginald Harrison, 6 Lower Berkeley Street, W. The travelling arrangements have been undertaken by Messrs. Cook and Sons.

The Committee of the West Wales branch of the National Association for the Prevention of Consumption, as £6,000 was available with which to begin building operations, decided to proceed at once with the work and secure plans for a Sanatorium.

A letter has been sent by Mr. Thos. Bowden Green, on behalf of the Street Noise Abatement Committee, to the Home Secretary, asking for increased powers to deal with unnecessary noises in business and residential localities. Medical testimony is quoted in support of the endeavours of the Committee.

King Edward's Hospital Fund has received £4,500 from the executor of the late Ch. H. Lear, and £1,000 from the executors of the late Rev. J. A. Price-Jones; and the executors of the late Miss M. F. Hasker have announced their intention to transfer to the trustees of the Fund £10,000, on condition that the amount is retained as capital by the Fund. This, with Mr. H. W. Marshall's gift of land, worth £12,000 now available, completes the sum required, bringing the permanent income of the Fund to £50,000 per annum.

In his evidence before the Royal Commission on the Care and Control of the Feeble-Minded, Dr. Caldecott, medical superintendent of the Earlswood Asylum, said that the various grades of "congenitally mentally deficient persons" practically required some form of permanent lifelong control, and he suggested that it was not necessary or advisable that there should be legislation for each individual grade. "Does it not appear," he asked, "that as a nation we shall make ourselves ridiculous if we keep on adding new Acts of Parliament for each supposed new grade to which well-meaning philanthropists choose to give a name?"

With a view to checking the abuse of charity, the Swansea General Hospital now require that patients shall sign on their letter of admission a declaration that they are unable to pay for private treatment. It is doubtful whether such a method will be effectual apart from adequate inquiry, but it is a step in the right direction and should help to educate people to understand that hospitals are not intended to relieve them of liabilities they are able to meet. A local paper commenting on this new departure at the Swansea Hospital suggests that one cause which helps to populate the hospitals by persons able to pay for private attendance is, that in calling in a medical man they are incurring an indefinite liability. It is the doctor who regulates the number of his visits, followed by an account drawn out "To medical attendance," without details. This arrangement does not fit in either with the feelings or condition of the majority of the poor, and they often incur the pauperising habit of depending on the hospital rather than incur an expense they cannot gauge. If a different *modus operandi* were to become customary it is not improbable that both the charities and medical men would benefit.

